# Secular Trends in Sugar-Sweetened Beverage Consumption Among Adults, Teens, and Children: The California Health Interview Survey, 2011–2018

**DOI:** 10.5888/pcd18.200399

**Published:** 2021-02-18

**Authors:** Matthew M. Lee, Emily Altman, Kristine A. Madsen

**Affiliations:** 1 Harvard T.H. Chan School of Public Health, Department of Nutrition, Boston, Massachusetts; 2 University of California, Berkeley, Berkeley, California

## Abstract

Consumption of sugar-sweetened beverages (SSBs) contributes to adverse health outcomes and excess health care spending. To provide context for ongoing work assessing the impact of public health strategies, including SSB excise taxes, we used data from the California Health Interview Survey from 2011–2018 to estimate trends in beverage consumption among adults, teens, and children overall and by education, race/ethnicity, and family income. We found reductions in the annual prevalence and frequency of soda consumption across all age groups and heterogeneous increases in the consumption of fruit drinks among adults and children. Surveillance of beverage consumption trends will continue to strengthen and improve the ability of researchers and policy makers to effectively improve population health.

**Summary**

**What is already known on this topic?**

Sugar-sweetened beverage (SSB) consumption contributes to excess weight gain and cardiometabolic disease risk, and strategies to reduce consumption have been implemented in the form of excise taxes in the United States

**What is added by this report?**

We present estimates for trends in soda and fruit drink consumption among adults, teens, and children in California between 2011–2018 to provide context for researchers and policy makers evaluating SSB consumption interventions.

**What are the implications for public health practice?**

SSB consumption, particularly of fruit drinks among adults and children, remains a persistent problem in California despite recent declines in soda consumption. Interventions to curb consumption must consider the possible unintended substitution of popular SSBs for other beverages.

## Objective

Consumption of sugar-sweetened beverages (SSBs) is associated with adverse health outcomes, including type 2 diabetes, cardiovascular disease, and dental caries (1). Although SSB intake declined in the United States from 2003 through 2016 (2), state-level surveillance is necessary for local or state-level policy makers interested in reducing consumption and health disparities in their communities (3). We previously reported on trends in soda consumption among adults in California from 2011 through 2016 (4). However, recent statewide trends in consumption of fruit and sports drinks and trends among teens and children are still unknown. Therefore, we sought to present a comprehensive and expanded analysis of trends in SSB consumption (soda and fruit drink) and water consumption in California from 2011 through 2018 among adults, teens, and children.

## Methods

By using data from the 2011–2018 cycles of the California Health Interview Survey (CHIS), we estimated beverage consumption frequency and prevalence among adults (≥18 y), teens (aged 12–17 y), and children (aged 0–11 y). Frequency was defined as the number of servings (eg, times, cans, glasses) consumed per unit time (ie, per week or day). Prevalence was defined as the proportion who consumed with any nonzero frequency during the same period. Consumption was reported directly by adults and teens and by a parent or guardian for children. CHIS survey weights allow for state-level inference (4). We identified questions related to beverage consumption and limited analyses to those asked in 2013 or earlier and again in 2017 or 2018, including soda (all ages), fruit drinks (adults/children); fruit/sports drinks (teens), and water (adults/teens). (More details on survey methods are available in the online Supplement available at https://madsenresearch.berkeley.edu.) For example, adults were asked: “During the past week, how often did you drink regular soda or pop that contains sugar? Do not include diet soda.” We did not compute estimates of consumption frequency or prevalence when data were not available. We converted reported adult consumption frequency of sweetened fruit drink from times per month to times per week by dividing the raw variable by 4.

Generalized linear models (GLMs) were conducted by using the adult, teen, and child data sets provided by CHIS. For primary analyses, we used log-Poisson models to obtain estimates of absolute consumption prevalence and changes in prevalence over time. In secondary analyses, we used log-gamma GLMs to obtain estimates of consumption frequency. Gamma-family GLMs were used to account for the right-skewedness, nonnegative nature, and presence of nonconsumers in beverage consumption data. We applied the jackknife method to replicate weights for variance estimation to obtain state-representative results and adjusted for yearly differences in race/ethnicity, sex, age, low family income (<200% of the federal poverty level, all ages), education (adults), nativity (adults), and survey language (teens and children), and explored heterogeneity by race/ethnicity (all ages), high school education (indicator for <high school diploma or general equivalency degree, adults), and family income (teens and children). Racial/ethnic categories were based on available variables in the CHIS public use files (Table). Adjusted marginal means were computed after each regression model.

**Table:** Descriptive Statistics (Unweighted Sample Sizes and Proportions) for Adults, Teens, and Children, by Year, California Health Interview Survey, 2011–2018

Characteristic	2011	2012	2013	2014	2015	2016	2017	2018	*P* a
**Adults aged ≥18 y**									
**Sex**									
Male	9,491 (42.0)	8,357 (41.1)	8,529 (41.2)	7,889 (40.4)	9,029 (42.9)	9,307 (44.2)	9,317 (44.1)	9,754 (46.1)	<.001
Female	13,089 (56.0)	11,998 (58.9)	12,195 (58.8)	11,627 (59.6)	12,005 (57.1)	12,005 (57.1)	11,748 (55.8)	11,423 (53.9)	
**Age, y**									
18–29	2,733 (12.1)	1,973 (9.7)	1,924 (9.3)	1,423 (7.3)	2,802 (13.3)	2,802 (13.3)	2,773 (13.1)	2,845 (13.4)	<.001
30–39	2,241 (9.9)	2,113 (10.4)	1,701 (8.2)	1,493 (7.7)	2,110 (10.0)	2,145 (10.2)	2,134 (10.1)	2,045 (9.7)	
40–49	3,178 (14.1)	3,176 (15.6)	2,655 (12.8)	2,227 (11.4)	2,502 (11.9)	2,442 (11.6)	2,364 (11.2)	2,297 (10.8)	
50–59	4,596 (20.4)	4,149 (20.4)	4,139 (20.0)	3,806 (19.5)	3,891 (18.5)	3,688 (17.5)	3,599 (17.0)	3,422 (16.2)	
≥60	9,832 (43.5)	8,944 (43.9)	10,305 (49.7)	10,567 (54.2)	9,729 (46.3)	9,978 (47.4)	10,283 (48.6)	10,568 (49.9)	
**Race/ethnicity**									
NH African American	1,095 (4.9)	902 (4.5)	979 (4.7)	785 (4.0)	1,217 (5.8)	1,024 (4.9)	1,045 (5.0)	1,151 (5.5)	<.001
NH White	14,471 (64.3)	11,272 (55.6)	13,324 (64.5)	12,319 (63.4)	12,456 (59.5)	11,196 (53.2)	13,258 (63.0)	12,368 (58.7)	
NH Asian/Alaska Native/mixed	2,379 (10.6)	3,165 (15.6)	2,147 (10.4)	2,549 (13.1)	2,310 (11.0)	3,412 (16.3)	1,997 (9.5)	2,830 (13.4)	
Hispanic	4,555 (20.2)	4,951 (24.4)	4,203 (20.4)	3,793 (19.5)	4,959 (23.7)	5,326 (25.4)	4,756 (22.5)	4,709 (22.4)	
**Education**									
<High school diploma	2,225 (9.9)	2,886 (14.2)	2,112 (10.2)	2,114 (10.8)	2,289 (10.9)	2,465 (11.7)	1,750 (8.3)	1,718 (8.1)	<.001
High school diploma or GED	5,166 (22.9)	4,567 (22.4)	4,370 (21.1)	4,228 (21.7)	4,806 (22.9)	4,919 (23.4)	4,354 (20.6)	4,410 (20.8)	
Some college	6,358 (28.2)	5,316 (26.1)	5,998 (28.9)	5,466 (28.0)	5,454 (25.9)	5,353 (25.4)	5,873 (27.8)	5,995 (28.3)	
≥College degree	8,831 (39.1)	7,586 (37.3)	8,244 (39.8)	7,708 (39.5)	8,485 (40.3)	8,318 (39.5)	9,176 (43.4)	9,054 (42.8)	
**Family income, % FPL**									
0–99	2,898 (12.8)	3,374 (16.6)	2,555 (12.3)	2,753 (14.1)	3,343 (15.9)	3,493 (16.6)	2,771 (13.1)	2,916 (13.8)	<.001
100–199	3,943 (17.5)	3,971 (19.5)	3,706 (17.9)	3,699 (19.0)	3,774 (17.9)	3,896 (18.5)	3,457 (16.3)	3,728 (17.6)	
200–299	3,254 (14.4)	2,854 (14.0)	3,037 (14.7)	2,711 (13.9)	2,834 (13.5)	2,699 (12.8)	2,831 (13.4)	2,921 (13.8)	
≥300	12,485 (55.3)	10,156 (49.9)	11,426 (55.1)	10,353 (53.0)	11,083 (52.7)	10,967 (52.1)	12,094 (57.2)	11,612 (54.8)	
**Nativity**									
US-born	17,743 (78.6)	14,058 (69.1)	16,320 (78.8)	14,875 (76.2)	16,158 (76.8)	15,422 (73.3)	17,089 (80.8)	16,814 (79.4)	<.001
Non–US-born	4,837 (21.4)	6,297 (30.9)	4,404 (21.3)	4,641 (23.8)	4,876 (23.2)	5,633 (26.8)	4,064 (19.2)	4,363 (20.6)	
**Teens aged 12–17 y**									
**Sex**									
Male	664 (49.7)	705 (48.2)	631 (52.5)	558 (53.0)	387 (51.3)	453 (53.9)	232 (51.8)	225 (52.1)	.12
Female	671 (50.3)	759 (51.8)	570 (47.5)	494 (47.0)	367 (48.7)	387 (46.1)	216 (48.2)	207 (47.9)	
**Age, y**									
12	213 (16.0)	219 (15.0)	154 (12.8)	166 (15.8)	121 (16.1)	127 (15.1)	75 (16.7)	65 (15.1)	.38
13	209 (15.7)	253 (17.3)	180 (15.0)	177 (16.8)	130 (17.2)	150 (17.9)	70 (15.6)	87 (20.1)	
14	221 (16.6)	263 (18.0)	207 (17.2)	170 (16.3)	143 (19.0)	140 (16.7)	76 (17.0)	74 (17.1)	
15	227 (17.0)	260 (17.8)	215 (17.9)	177 (16.8)	114 (15.1)	140 (16.7)	81 (18.1)	70 (16.2)	
16	222 (16.6)	247 (16.9)	208 (17.3)	185 (17.6)	116 (15.4)	141 (16.8)	81 (18.1)	79 (18.3)	
17	243 (18.2)	222 (15.2)	237 (19.7)	177 (16.8)	130 (17.2)	142 (16.9)	65 (14.5)	57 (13.2)	
**Race/ethnicity**									
Latino	358 (26.8)	490 (33.5)	339 (28.2)	313 (29.8)	312 (41.4)	371 (44.2)	152 (33.9)	148 (34.3)	<.001
NH Asian	113 (8.5)	150 (10.3)	82 (6.8)	109 (10.4)	59 (7.8)	91 (10.8)	30 (6.7)	32 (7.4)	
NH White	620 (46.4)	533 (36.4)	525 (43.7)	415 (39.5)	289 (38.3)	285 (33.9)	211 (47.1)	196 (45.4)	
Other/multiple	244 (18.3)	291 (19.9)	255 (21.2)	215 (20.4)	94 (12.5)	93 (11.1)	55 (12.3)	56 (13.0)	
**Survey language**									
English	1,250 (93.6)	1,347 (92.0)	1,146 (95.4)	1,004 (95.4)	605 (80.2)	623 (74.2)	397 (88.6)	402 (93.1)	<.001
Other	85 (6.4)	117 (8.0)	55 (4.6)	48 (4.6)	149 (19.8)	217 (25.8)	51 (11.4)	30 (6.9)	
**Family income, % FPL**									
0–99	226 (16.9)	344 (23.5)	220 (18.3)	217 (20.6)	169 (22.4)	186 (22.1)	67 (15.0)	46 (10.7)	<.001
100–199	276 (20.7)	364 (24.9)	262 (21.8)	244 (23.2)	192 (25.5)	183 (21.8)	75 (16.7)	78 (18.1)	
200–299	171 (12.8)	175 (12.0)	143 (11.9)	134 (12.7)	88 (11.7)	99 (11.8)	50 (11.2)	42 (9.7)	
≥300	662 (49.6)	581 (39.7)	576 (48.0)	457 (43.4)	305 (40.5)	372 (44.3)	256 (57.1)	266 (61.6)	
**Children aged 0–11 y**									
**Sex**									
Male	1,818 (52.1)	1,977 (51.4)	1,493 (51.1)	1,352 (52.2)	1,092 (50.6)	1,109 (51.9)	842 (52.6)	817 (51.5)	.92
Female	1,670 (47.9)	1,869 (48.6)	1,427 (48.9)	1,240 (47.8)	1,065 (49.4)	1,027 (48.1)	758 (47.4)	769 (48.5)	
**Age, y**									
0	240 (6.9)	215 (5.6)	157 (5.4)	135 (5.2)	168 (7.8)	186 (8.7)	94 (5.9)	103 (6.5)	<.001
1	265 (7.6)	245 (6.4)	170 (5.8)	149 (5.8)	166 (7.7)	167 (7.8)	107 (6.7)	106 (6.7)	
2	308 (8.8)	265 (6.9)	194 (6.6)	168 (6.5)	163 (7.6)	167 (7.8)	139 (8.7)	116 (7.3)	
3	276 (7.9)	302 (7.9)	222 (7.6)	187 (7.2)	171 (7.9)	166 (7.8)	113 (7.1)	89 (5.6)	
4	345 (9.9)	346 (9.0)	243 (8.3)	191 (7.4)	215 (10.0)	193 (9.0)	136 (8.5)	115 (7.3)	
5	307 (8.8)	352 (9.2)	225 (7.7)	238 (9.2)	197 (9.1)	209 (9.8)	107 (6.7)	123 (7.8)	
6	252 (7.2)	291 (7.6)	245 (8.4)	201 (7.8)	138 (6.4)	155 (7.3)	129 (8.1)	102 (6.4)	
7	281 (8.1)	307 (8.0)	258 (8.8)	222 (8.6)	159 (7.4)	128 (6.0)	118 (7.4)	143 (9.0)	
8	246 (7.1)	342 (8.9)	268 (9.2)	231 (8.9)	174 (8.1)	142 (6.7)	138 (8.6)	132 (8.3)	
9	284 (8.1)	409 (10.6)	281 (9.6)	285 (11.0)	157 (7.3)	170 (8.0)	158 (9.9)	158 (10.0)	
10	341 (9.8)	363 (9.4)	299 (10.2)	309 (11.9)	227 (10.5)	236 (11.1)	183 (11.4)	185 (11.7)	
11	343 (9.8)	409 (10.6)	358 (12.3)	276 (10.7)	222 (10.3)	217 (10.2)	178 (11.1)	214 (13.5)	
**Race/ethnicity**									
Latino	963 (27.6)	1,343 (34.9)	826 (28.3)	742 (28.6)	969 (44.9)	1,076 (50.4)	379 (23.7)	362 (22.8)	<.001
NH Asian	346 (9.9)	498 (13.0)	273 (9.4)	337 (13.0)	139 (6.4)	186 (8.7)	110 (6.9)	143 (9.0)	
NH Black	153 (4.4)	151 (3.9)	116 (4.0)	94 (3.6)	120 (5.6)	91 (4.3)	84 (5.3)	100 (6.3)	
NH White	1,677 (48.1)	1,450 (37.7)	1,399 (47.9)	1,141 (44.0)	757 (35.1)	624 (29.2)	799 (49.9)	753 (47.5)	
Other/multiple	349 (10.0)	404 (10.5)	306 (10.5)	278 (10.7)	172 (8.0)	159 (7.4)	228 (14.3)	228 (14.4)	
**Survey language**									
English	2,803 (80.4)	2,545 (66.2)	2,292 (78.5)	1,966 (75.9)	1,856 (86.1)	1,636 (76.6)	1,384 (86.5)	1,381 (87.1)	<.001
Other	685 (19.6)	1,301 (33.8)	628 (21.5)	626 (24.2)	301 (14.0)	500 (23.4)	216 (13.5)	205 (12.9)	
**Family income, % FPL**									
0–99	671 (19.2)	863 (22.4)	563 (19.3)	526 (20.3)	521 (24.2)	563 (26.4)	272 (17.0)	241 (15.2)	<.001
100–199	725 (20.8)	909 (23.6)	577 (19.8)	555 (21.4)	479 (22.2)	504 (23.6)	307 (19.2)	306 (19.3)	
200–299	451 (12.9)	455 (11.8)	377 (12.9)	351 (13.5)	255 (11.8)	228 (10.7)	211 (13.2)	224 (14.1)	
≥300	1,641 (47.1)	1,619 (42.1)	1,403 (48.1)	1,160 (44.8)	902 (41.8)	841 (39.4)	810 (50.6)	815 (51.4)	

Abbreviations: GED, general equivalency degree; NH, non-Hispanic; FPL, federal poverty level.

a *P* values determined by using 2-tailed χ^2^ tests. All values are expressed as number (%) and are unweighted.

In sensitivity analyses, we used categorical representations of year to qualitatively assess the validity of the continuous representation in our primary results. All analyses were conducted by using Stata/MP version 16.1 (StataCorp LLC). This research was considered exempt from review by the University of California Berkeley Committee for the Protection of Human Subjects.

## Results

We found significant differences over time in the distributions of all covariates except sex (for teens and children) and teen age (Table). Adult CHIS respondents were older in 2018 compared with 2011, less likely to identify as non-Hispanic White, and more likely to have a college degree or higher. Compared with teen respondents in 2018, those in 2011 were more likely to identify as Latino and to have a family income that was at 300% of the FPL or higher. Last, child respondents in 2018 were older, more likely to identify as non-Hispanic Black or other or multiple race/ethnicity, less likely to have completed the survey in a non-English language, and more likely to have higher family incomes.

By using continuous representations of time, soda consumption prevalence declined by 1.01% (95% CI, 0.33%–1.68%) annually among adults, 4.24% (−0.98% to 9.20%) among teens, and 7.60% (0.20%–14.45%) among children (Figure). Teens alone experienced yearly reductions in soda consumption frequency of 6.50% (2.06%–10.75%). 

**Figure:**
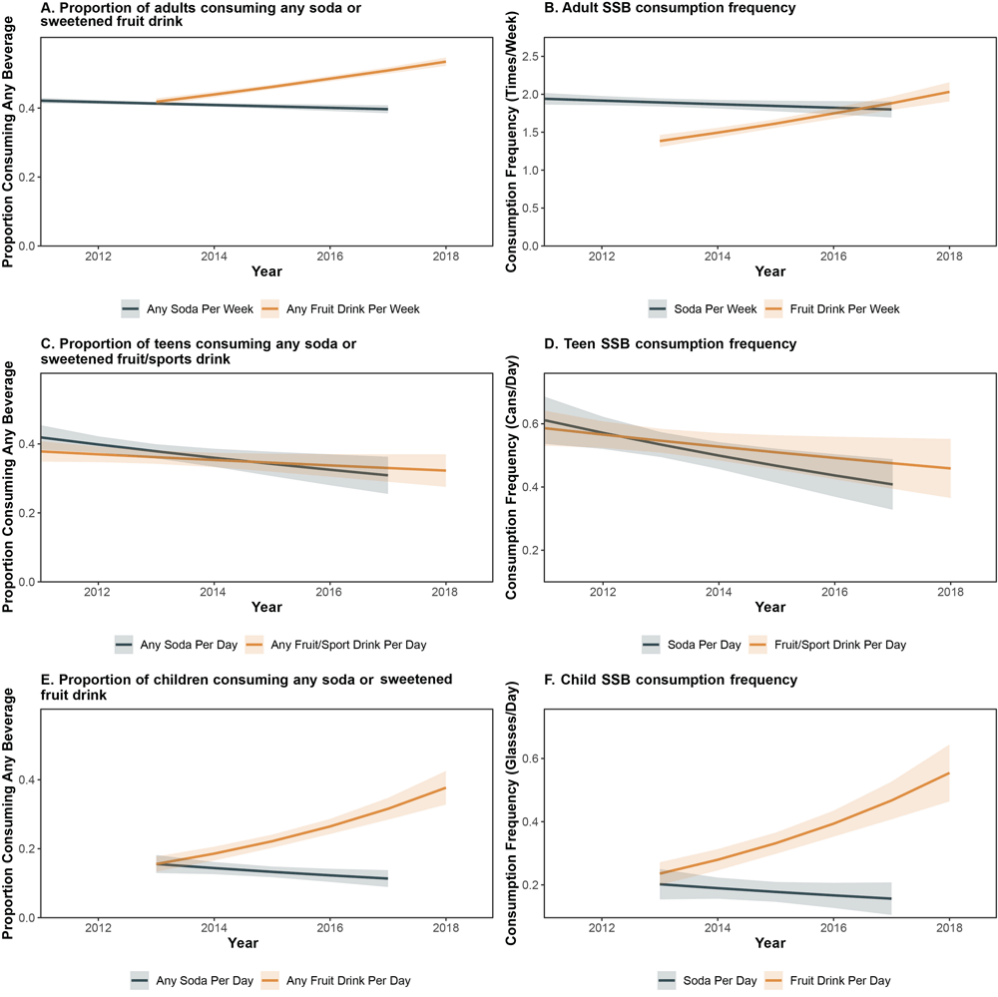
Trends in proportion of Californians consuming various beverages and the amount consumed from 2011 through 2018, by age group and beverage type, California Health Interview Survey, 2011–2018. Trends of soda and fruit drink consumption are shown for adults (aged ≥18 y), of soda and fruit or sport drink consumption for teens (aged 12–17 y), and for soda and fruit drink consumption for children (aged 0–11 y). Abbreviation: SSB, sugar-sweetened beverage.

Inspection of trends by categorical year confirmed the validity of log-linear relationships between consumption and time, except for adult soda consumption frequency (Online Supplement, eFigure 1). Adult soda consumption prevalence was significantly higher in 2011 than in all subsequent years, and soda consumption frequency was greater in 2011 than in all years but 2015 or 2016. Prevalence of adult soda consumption declined among respondents with at least a high school education for all years compared with 2011 (*P* < .01 for all but 2012) but did not change for adults with below high school education. No other significant differences were found in soda consumption trends by race/ethnicity or family income. 

Conversely, consumption of sweetened fruit drinks increased in adults and children from 2013 through 2018. Annual growth in adult consumption prevalence was greater for White participants (5.8%) compared with Black or Latinx participants (0.8% and 4.0%, respectively; *P* < .001 for interaction). Child fruit drink consumption prevalence increased 19.4% annually (95% CI, 14.3%–24.6%), from 15.6% in 2013 to 37.7% in 2018. Increases were greater among children at or below 200% FPL compared with their wealthier counterparts for fruit drink consumption frequency (24.8% and 13.4%, respectively, *P = *.03 for interaction) and for prevalence (24.3% and 14.4% respectively, *P = *.03 for interaction).

Increases in water consumption were statistically but not clinically significant because of high levels of consumption reported at all time points. (All data are available at https://madsenresearch.berkeley.edu.)

## Discussion

We found annual reductions in the proportion of soda consumers across age levels in CHIS data for 2011–2018. Teens alone also exhibited consistent declines in the amounts of soda and sweetened fruit or sport drinks consumed. Detecting consumption-trend heterogeneity among teens was challenging, because this group’s sample sizes were smaller than those of adults or children; however, qualitative assessment suggested that potential differences by race/ethnicity or family income were small (eFigures 1.2.1 and 1.2.2). Decreases in children’s soda consumption were also independent of race/ethnicity and family income.

Levels of SSB consumption remained generally higher among African-American and Latinx respondents and for adults without a high-school diploma. This pattern was established previously among adults and children in CHIS (4,5) and was also seen in the overall US population (6), and it may reflect persisting disparities in income, the food environment, health resources, and advertising (7). Also of concern were large increases in consumption of sweetened fruit-drinks among adults and children; these increases may indicate replacement of soda with fruit drinks and highlights the importance of public-health messaging when promoting SSB reductions (8). Consistent with previous work in CHIS, fruit drink intake was higher among children living in low-income households (9). Compared with US estimates of beverage consumption from 2011 through 2014, soda consumption prevalence in California was similar among teens, higher among adults, and lower among children. Prevalence of fruit drink consumption in California was higher for adults and teens but lower for children compared with that at the national level (6).

Our study’s limitations included the repeated cross-sectional design of CHIS, the possibility of unmeasured confounding that could otherwise obscure beverage consumption trends, and measurement error due to recall or social desirability bias. CHIS does not assess beverage intake portion size and therefore cannot be used to draw conclusions related to trends in consumption volume. We conducted yearly analyses to assess trends in intake, but future work should evaluate sensitivity of results when pooling across multiple years of data instead. Last, CHIS public use files did not include a separate racial/ethnic category for African American teens, and additional research on health disparities during this critical age period should consider the feasibility of the restricted data.

SSB excise taxes, effective in reducing consumption and significantly cost-saving over the life-course (10–13), have been implemented in 4 California cities: Berkeley (2015), San Francisco (2018), Oakland (2017), and Albany (2017). As additional strategies are proposed and implemented, continued surveillance of state-wide trends will strengthen the ability to assess their impact and identify areas for improvement.
